# The effect of financial crises on deforestation: a global and regional panel data analysis

**DOI:** 10.1007/s11625-021-01086-8

**Published:** 2022-01-29

**Authors:** Alexander S. Antonarakis, Lucia Pacca, Andreas Antoniades

**Affiliations:** 1 School of Global Studies, University of Sussex, Falmer, Brighton, BN1 9QJ UK; 2 Center for Vulnerable Populations, University of California, San Francisco, USA

**Keywords:** Deforestation, Financial crises, Environmental sustainability, Panel data analysis, Deforestation drivers, Forest cover datasets

## Abstract

Managing our transition to sustainability requires a solid understanding of how conditions of financial crisis affect our natural environment. Yet, there has been little focus on the nature of the relationship between financial crises and environmental sustainability, especially in relation to forests and deforestation. This study addressed this gap by providing novel evidence on the impact of financial crises on deforestation. A panel data approach is used looking at Global Forest Watch deforestation data from > 150 countries in > 100 crises in the twenty-first century. This includes an analysis of crises effects on principle drivers of deforestation; timber and agricultural commodities—palm oil, soybean, coffee, cattle, and cocoa. At a global level, financial crises are associated with a reduction in deforestation rates (− 36 p.p) and deforestation drivers; roundwood (− 6.7 p.p.), cattle (− 2.3 p.p.) and cocoa production (− 8.3 p.p.). Regionally, deforestation rates in Asia, Africa, and Europe decreased by − 83, − 43, and 22 p.p, respectively. Drivers behind these effects may be different, from palm oil (− 1.3 p.p.) and cocoa (− 10.5 p.p.) reductions in Africa, to a combination of timber (− 9.5 p.p) and palm oil in Asia. Moreover, financial crises have a larger effect on deforestation in low-income, than upper middle- and high-income countries (− 51 vs − 39 and − 18 p.p. respectively). Using another main dataset on yearly forest cover—the ESA-Climate Change Initiative—a picture arises showing financial crises leading to small global decreases in forest cover (− 0.1 p.p.) with a small agricultural cover increase (0.1 p.p). Our findings point to financial crises as important moments for global deforestation dynamics. Yet, to consolidate benefits on decreasing deforestation, governments need to enhance their sustainable forest management during crisis periods rather than let it slip down national agendas. Finally, to achieve the SDGs related to forests, better global forest cover datasets are needed, with better forest loss/gain data, disturbance history, and understanding of mosaicked landscape dynamics within a satellite pixel.

## Introduction

Financial crises have been recognized by the UN as a real threat to the global development agenda (UNCTAD 2015; WESS 2017). Forty percent of low-income countries were facing significant challenges in servicing their debt already before the COVID-19 pandemic crisis (IMF 2018). The unprecedented pandemic impact has seen sharp falls in livelihoods and GDP globally. Advanced economies are projected to return to their pre-pandemic output level in 2024; emerging and developing economies (excluding China) are projected to still be 5.5% below their pre-pandemic GDP level in 2024, while the output of low-income economies is projected to continue declining at least up to 2024 (IMF 2021). The effects of financial crises are often severe and widespread and go well beyond the economic performance of countries (Antoniades et al. 2020; Antoniades et al. 2022). Given the linkages between the economy and the environment (Shafik and Bandyopadhyay 1992; Lambin and Meyfroidt 2011; Cuaresma et al. 2017), it follows that a shock in the former should affect the latter. Economic globalization has seen nations expand their agricultural land and output at the expense of natural and forested landscapes (Ramankutty et al. 2008; Lambin and Meyfroidt 2011). Global trade is an important component of this, redistributing environmental impacts associated with natural resources, carbon emissions, and agricultural commodities, sometimes from higher to lower income countries (Srinivasan et al. 2008). Yet, the impacts or shocks of financial crises on the environment have not been well defined. For example, Bowen and Stern (2010) make the case that a demand-induced economic downturn could provide a great opportunity to step up public spending on environmental policies, yet recent evidence has shown that environmental protection may be weakened during a financial crisis (Lekakis and Kousis 2013; Gaveau et al. 2009; Botetzagias et al. 2018). Additionally, evidence on measuring financial crisis impacts on the environment and environmental protection has so far been neglected (Burns and Tobin 2016; Botetzagias et al. 2018; for a recent literature review, see Antoniades and Antonarakis 2022). In a previous study, Pacca et al. (2020) investigated the effects of financial crisis on air pollutant emissions and found significant short-term global decreases in CO_2_, SO_2_, and NO_x_ by 2.6, 1.8, and 1.7 p.p., respectively.

In this paper, we focus on the relationship between financial crises and deforestation. Deforestation is of principal concern globally with explicit SDG targets (SDG 15.2) pushing for the halt to deforestation, and initiatives such as the UN Strategic Plan for Forests 2017–2030, the UN program for Reducing Emissions from Deforestation and forest Degradation, the Zero-Deforestation Commitments for producers and traders (Humphreys et al. 2019), the New York Declaration on Forests, the UN Decade 2021–2030 on Ecosystem Restoration all calling for an end to deforestation and forest degradation. There are many drivers of deforestation linked to national economic development. Agricultural expansion is the principal driver of deforestation accounting for 80% of forested land-cover change (FAO 2016a), with large-scale commercial agriculture accounting for more than half of this in developing countries (Hosonuma et al. 2012). Agricultural commodities include soybean and cattle-ranching in South America, oil palm plantations in South-East Asia, as well as cocoa, banana, and coffee among others. The top three commodities alone—soybean, beef, oil palm (Pendrill et al. 2019)—are globally worth over 110 billion USD in exports annually (FAO 2016b). In fact, commodity-driven agriculture is the largest driver of deforestation in tropical South America and South-East Asia, whereas shifting agriculture is the main driver of forest loss in Africa with large minority contributions in South America and South-East Asia (Curtis et al. 2018). Urban expansion, infrastructure, and mining are also large contributors to deforestation in tropical and subtropical countries (DeFries et al. 2010; Hosonuma et al. 2012).

Beyond the tropics and subtropics, forestry, in managed forests or plantation, and wildfires, with no subsequent human conversion to crops, are the main drivers of forest loss (although wood products are also a significant driver of tropical forest loss). Forestry is the principal cause of forest loss in Europe and North America, with large forestry sectors existing in Russia, East Asia, Australia, and southern South America (Curtis et al. 2018). Wildfires are dominant in Russia, Canada, and Australia. The global forestry product industry accounts for 250 billion USD exports annually (FAO 2018a), but illegal timber can account for over 70% of the income of countries’ timber exports (World Bank 2012).

Considering financial crises, existing studies have found different and often contradictory channels underlying the relationship between crises, and forest loss and deforestation. For example, financial crises resulted in intensification of forest protection initiatives in the Brazilian Amazon, promoted by NGOs, during the crises years 1998–2000, as well as cutting resources to environmentally damaging activities such as road-building (Kasa and Naess 2005). On the contrary, cut in resources in forest management and conservation has been blamed for an increase in deforestation as a result of financial crises in South-East Asia (Dauvergne 1999; Siddiqi 2000; Pagiola 2001) and Greece (Lekakis and Kousis 2013). The reduction in government expenditures in forest protection was manifested in some cases as a shrinking of forest rangers or law enforcement to protect forests, e.g., Gaveau et al. (2009) found that the 1997–1998 crisis brought about an 18-fold increase in deforestation in Sumatra attributed mainly to a weakening of law enforcement. The current pandemic is also seeing increased logging activity attributed to reduced enforcement (Fair 2020).

Agriculture is often seen by governments as a way to drag countries out of financial crises through a decrease in unemployment, higher social and political stability, and more export opportunities (Sunderlin 1999). Indeed, Dauvergne (1999) found that agriculture was expanded in East-Asia during the 1997–1998 crisis as a way to drag countries out of the crisis and compensate for households’ shortfall in income. Crises can also induce a change in prices of agricultural goods, which encourages production of some to be expanded and other to be decreased. For example, the price of palm oil increased subsequent to the 1997 East-Asian crisis and 2008/2009 Global Financial Crisis resulting in an expansion of cultivated areas of palm in Indonesia (Pagiola 2001; Maxton-Lee 2018). Shifting agricultural practices may also impact on forests. For example, following the Global Financial Crisis in late 2008, staff working for logging companies in Cameroon were made redundant, resulting in an increase in poaching and slash and burn agriculture (Sayer et al. 2012). These increases in demand for agricultural land may result in urban-to-rural and rural-to-rural migration toward natural land and forest frontiers (Pagiola 2001; Carr 2009), although other studies have noted a rural-to-urban migration due to a decline in mining, volatile food prices, and timber activities (UNECA 2009; Tieguhong et al. 2009).

Timber, as a major trade commodity, for export and fuelwood is also affected during economic crises (Nilsson 2009; Presas 2009; FAO 2020) for instance, via crashes in the property market and downturns in the construction industry (Busch and Ferretti-Gallon 2017). Notably, timber production and trade suffered during the 2008/09 global recession (Nilsson 2009; Eurostat 2019). Decreases in national and international timber demand during a crisis can result in lower production (Dauvergne 1999; Elliott 2011). According to Dauvergne (1999), this decrease in logging activities did not necessarily produce great environmental benefits. Indeed, countries in South-East Asia moved into more profitable and equally environmentally harmful activities, such as rubber plantations and palm oil. Similar channels have been also identified by Elliott (2011) who found that although demand for timber in Indonesia contracted during the crisis leading to a reduction in forest exploitation, this outcome was compensated by an intensification of illegal timber activities. Illegal collection of forest products to generate income (Gross et al. 2014) and energy (Pagiola 2001; Lekakis and Kousis 2013) can be triggered by a collapse in the economy or an increase in fuel prices. Some of the channels identified relating financial crisis and forest loss are shown in Table 1.

**Table 1:** Main channels: financial crises and forest loss

Decrease in deforestation	Increase in deforestation
Intensification of forest protection initiatives, promoted by NGOs, during the crises year (Kasa and Naess 2005)	Increased collection of forest products to generate energy (Pagiola 2001; Lekakis and Kousis 2013)
Cut in resources allocated to environmentally damaging activities, such as large infrastructure projects (e.g. road-building, mines, hydroelectric dams) (Kasa and Naess 2005; Pagiola 2001; Laurance et al. 2015)	Cut in resources for forest management and conservation (Siddiqi 2000; Kasa and Naess 2005; Pagiola 2001; Fair 2020), including capacity to deal with fires (Lekakis and Kousis 2013)
Decrease in national and international timber demand, resulting in lower production (Dauvergne 1999; Elliott 2011; FAO 2020)	Increase in agricultural activities compensating for households’ shortfall in income (Dauvergne 1999; Pagiola 2001)
Rural-to-urban migration due to declines in timber demand, redundancies in mining and volatile food prices, resulting in less pressure on natural land (UNECA 2009; Tieguhong et al. 2009)	Increase in prices of some commodities during the crisis years, with resulting expansion of cultivated area (e.g., palm oil in Indonesia after 1997) (Pagiola 2001)
Commodity price fluctuations, especially in the form of price decreases (e.g., palm oil and timber) (Pagiola 2001; Maxton-Lee 2018; Sulaksono and Widjanarko 2009)	Weakening of law enforcement to protect forests during the crisis years (Gaveau et al. 2009; Pagiola 2001), with resulting intensification in illegal forest activities (Elliott 2011; Lekakis and Kousis 2013; Gross et al. 2014)
	Return, urban-to-rural, migration of workers who lose their jobs; and rural-to-rural migration toward forest frontiers (Pagiola 2001; Carr 2009)

The contradiction in these studies looking at the impact of financial crises on deforestation lies largely in that they are country-level case studies or regional assessments of deforestation. Each country may have different drivers of forest loss which may become exacerbated or differently affected during times of economic downturn. For instance, agricultural expansion during crises is given as a reason for increases in deforestation in Indonesia (Dauvergne 1999; Pagiola 2001), while a decline in timber demand resulted in lower forestry production in Indonesia (Dauvergne 1999; Elliott 2011); or intensification in forest protection was promoted during a financial crisis in the Brazilian Amazon (Kasa and Naess 2005), but conservation and forest management was cut in Southeast Asia during the Asian financial crisis (Siddiqi 2000; Kasa and Naess 2005; Pagiola 2001). Furthermore, in many of the case studies, assessments of the effect of financial crises on deforestation were not derived from statistical relationships.

This study seeks new evidence on the impact of financial crises on deforestation, advancing the current knowledge in four ways. First, we examine the relationship between financial crises and deforestation across countries in the global context moving beyond single country evaluations. This empirical analysis is based on yearly satellite-derived deforestation data from the Global Forest Watch (GFW) from 2001 to 2017 in more than 150 countries for over 100 crises events, drawing generalized global evidence of financial crisis effects on changes to forested land. We also examine heterogeneity in these effects across continental and national income groupings. Second, we investigate the financial crisis effect on two proximate drivers of global forest loss: agriculture commodity and forestry products. Agriculture is subdivided into agricultural land-cover change, and production of palm oil, soybean, coffee, cattle, and cocoa. Palm oil, soy, and beef alone can contribute to 76% of deforestation associated with agriculture (Brack et al. 2016), and in some cases, cattle, wood products, soybean, and palm oil together can contribute to more than a third of tropical deforestation (Persson et al. 2014). Third, we compare the two available yearly datasets of global deforestation and forest cover: the GFW and the European Space Agency Climate Change Initiative (ESA CCI). The ESA CCI estimates yearly forest cover changes from 1992 to 2015. This comparison will provide insight into financial crisis effects on these two key datasets, and the nature and quality of data available to help us meet the SDG goals related to forests.

## Method

### Data sources

Data on financial crises come from Laeven and Valencia (2018). The database includes three different types of crises: systemic banking crises, sovereign debt crises, and currency crises occurring between 1970 and 2017. Banking crises are defined if two conditions are met: signs of financial distress in the banking system and significant banking policy interventions. Currency crises are defined as a nominal depreciation of the country’s currency vis-à-vis the U.S. dollar of at least 30%, that is also at least 10 percentage points higher than the rate of depreciation in the year before. As for sovereign debt crises, these include episodes of sovereign debt default or restructuring. These three different types of crises are combined into one variable in this analysis with 103 crises over 165 countries (listed in Appendix Table 11) in the twenty-first century (concurrent with GFW data), and 239 crises between 1992 and 2017 (concurrent with ESA-CCI forest cover data).

Data on yearly deforestation are taken from the Global Forest Watch on forests with > 30% canopy cover (Hansen et al. 2013; GFW 2014). These public maps measure near-real-time (yearly) deforestation in hectares, derived from Landsat satellite observations. Data are available from 2001 onwards and over 165 countries, with raw data at a resolution of 30 m. Data on forest gain, available once from 2001 to 2012, were not used. In our analysis, we also use forest coverage from the ESA-CCI (Defourny et al 2017). These data measure forest covered area in hectares and are available yearly from 1992 to 2015. In addition to the longer time availability, this database provides a net forest cover change, accounting for not only forest losses, but also forest gains through, for example, reforestation initiatives or plantation growth. Therefore, it offers supplementary information with respect to the GFW data. Raw ESA CCI data from 1992 are provided at 300 m resolution for a number of land-cover types over the globe, and have been determined using satellites AVHRR, MERIS, SPOT-Vegetation, and PROBA-V. All forested land-cover types from the ESA-CCI (Defourny et al. 2017) were combined into one forested class—merging classes 50–90 and 160, 170 with small contributions from other classes (see FAOSTAT 2017).

Agricultural land from 1992 to 2015 was also taken from the ESA CCI, merging classes 10–40, including rainfed, irrigated, and mosaicked cropland (see FAOSTAT 2017). Data on yearly roundwood production in millions m^3^ per year are taken from the FAO (2018a, b) for 209 countries and dependencies and are available from 1961 to 2017. Roundwood production encompasses both industrial roundwood and wood fuel. Production of agricultural commodities common in tropical countries of palm oil, soybean, cattle, coffee, and cocoa in tons per year are available from FAOSTAT from 1992 to 2017 for 194 countries. Regarding the control variables, agricultural employment, trade openness, and urban population come from the World Bank’s World Development Indicators, while data on total primary energy use are taken from the Energy Information Administration (EIA). Table 2 shows all the dependent and independent variables used in this study.^1^

**Table 2:** Variables used in this study

Dependent variables	Units	Sample length	Number of countries	Source
Deforestation	1000 Ha	2001–2017	173	GFW
Forest Cover	1000 Ha	1992–2015	211	ESA CCI
Roundwood Production	Millions m^3^	1961–2017	209	FAO
Agricultural Production (palm oil, soybean, coffee, cattle, and cocoa)	Tons	1992–2017	194	FAOSTAT
Agricultural Cover	1000 Ha	1992–2015	211	ESA CCI

Independent and control variables				
Financial Crisis	Dummy var	1970–2017	165	Laeven and Valencia (2018)
Urban Population	% of total	1960–2017	213	World Bank
*per capita *Energy	Btu per cap	1980–2017	215	EIA
Trade openness	% of GDP	1960–2017	199	World Bank
Agricultural Employment	% of total	1991–2017	186	World Bank

### Econometric specification

To assess the impact of crises on the environmental variables of deforestation, forest cover change, roundwood production, agricultural commodity production, and agricultural cover change, we estimate the following empirical specification for Ordinary Least Squares (OLS) and Fixed Effects (F.E.):

1 $$y_{it} = \alpha_{it} + y_{it - 1} \; + \;crisis_{it} \; + \;X_{it} \; + \;c_{i} \; + \;\varepsilon_{it} ,$$

where *y* is our dependent variable, and for each separate model represents deforestation, forest cover change, roundwood production, agricultural commodity production *or* agricultural cover change, in country *i* and year *t*. *Crisis* is the financial crisis dummy variable, equal to one in years when country *i* is experiencing a crisis, and equal to zero in all the other years, and is a combination of all types of crises defined in Laeven and Valencia (2018). $X$ is a vector of control variables given in Table 2, and $\alpha$ is the constant term. *c*_*i*_ are unobserved time-invariant country effects, for example geographic, historical, and institutional conditions. Finally,$\varepsilon_{it}$ is the error term.

In this study, we also use the generalized method of moments (GMM) model

2 $$y_{it} = \alpha_{it} + y_{it - 1} \; + \;crisis_{it} \; + \;X_{it} \; + \;\varepsilon_{it} .$$

The use of the GMM is theorized by the dynamic panel data methodology developed by Arellano and Bond (1991). In particular, we adopt this approach to overcome the dynamic panel bias created by the inclusion of the lagged dependent variable ($y_{it - 1}$), which might generate autocorrelation between the predictor variables and the error term. The reason why we employ the GMM in addition to regular panel OLS and F.E. estimations is that the latter might lead to biased and inconsistent estimates as they do not control for this bias. The GMM estimator, suited for “small T, large N” panels, manages the endogeneity issue by instrumenting the lagged dependent variable and/or any other endogenous variables with the previous (second and further) lags, which are thought to be uncorrelated with the fixed effects (Roodman 2009). Furthermore, the GMM approach removes time-constant unobserved variables (*c*_*i*_) which may correlate with the dependent or control variables by implementing a first difference transformation (Arellano and Bond 1991; Arellano 2003).

All dependent and independent variables in Eqs. (1 and 2) are included as growth rate terms rather than level terms. Using growth rates allows for comparison and statistical inference of differently sized entities. Our dependent and independent variables considerably vary in size and unit of measurement (see Table 2), which would make it difficult to interpret beta coefficients if they were included in level.

Regarding control variables, we include the percentage of urban population over the total population, the level of trade openness, per capita energy consumption, and the level of agricultural employment. These selected control variables are similar to recent econometric single country and panel data analyses predicting deforestation (Tsurimi and Managi 2014; Ahmed et al. 2015; Maji 2017; Nathaniel and Bekun 2020), and are related to the determinants of forest loss. Growth in urban population could influence deforestation in several ways. On one side, the proximity of forested areas to large cities and the density of urban areas have been linked to higher deforestation (see, for example, Nelson and Hellerstein 1997; Cropper et al. 1997; De Fries et al. 2010). This mostly happens through the intensification of road building and construction, and the transition from subsistence agriculture to market-oriented agriculture to accommodate the needs of the growing population. On the other hand, increases in rural settlements may also be linked to pressures on forest ecosystems (Assunção and Rocha 2016).

Concerning trade openness, it can be related to increased exports of timber and agricultural commodities putting pressure on forests but can also allow imports which can reduce the incentive to deforest (Meyfroidt et al. 2010; Faria and Almeida 2016). Specifically, forests in more developed countries may benefit from trade openness at the expense of lesser developed countries where more environmentally damaging commodity production occurs (Tsurimi and Managi 2014). Energy consumption from environmentally damaging sources can have a negative impact on forests (Bawa and Dayanandan 1997; Ahmed et al. 2015). Renewable energy, on the contrary, has been shown to reduce pressure on forests (Ponce et al. 2021), although even green technology for renewables and sustainable infrastructure can put forests at risk from mining (Bradley 2020). Finally, agricultural employment consisting of agriculture, hunting, forestry, and fishing activities is linked to the development of the agricultural sector, and can be considered a proxy for the development stage of a country (FAO 2018b). Forest conversion to agriculture is more dominant among the mechanized and market dominated farmers rather than poorer subsidence farmers (Lambin and Meyfroidt 2011; Olanipekun et al. 2019).

## Results

### Financial crisis on deforestation from Gobal Forest Watch

Table 3 presents results on the effect of crises on forest loss, using deforestation from Global Forest Watch as the dependent variable. In column 1, an OLS specification is reported, where the only predictor variable is the financial crisis dummy. In column 2, we add the lagged dependent variable. All five models in Table 3, including the two first models without control variables, are included to demonstrate the robustness of our results (sign and magnitude of effect). The coefficient on the financial crisis indicator for column 1 and 2 is negative and statistically significant, and shows an average 42–45 percentage point (p.p.) decrease in deforestation in years when countries experience a crisis as compared to years when no crisis happens. When including our control variables (column 3), the coefficient on the financial crisis dummy slightly decreases, and becomes equal to − 0.34 (34 percentage points decrease in deforestation). However, its sign and significance does not change. The magnitude of our main coefficient of interest is confirmed by the F.E. and GMM specifications (columns 4 and 5), providing robustness of our results. The GMM results show that financial crises result in 36 percentage points decrease in deforestation using all countries in the period 2001–2017. These results are not affected by outliers, where the 5th and 95th percentiles of countries by forest cover were removed resulting in changes in decrease in deforestation rates by 6–7 p.p. less than standard errors reported below in Table 3, and still highly significant. Regarding the covariates, decreases in urban population and *per capita* energy growth and increases in trade growth are associated with decreases in deforestation. However, when using the GMM model, only the coefficient on urban population maintains its significance. The relationship between the 2008-09 Global Financial Crisis and global deforestation was also examined finding highly significant results of a deforestation decrease by 16–20 p.p. (see Table 13).

**Table 3:** Effect of financial crises on deforestation: global data

Dependent variable: Deforestation growth	(1)	(2)	(3)	(4)	(5)
	OLS	OLS	OLS	Fixed effects	GMM
Financial Crisis	− 0.424***	− 0.451***	− 0.344***	− 0.391***	− 0.362***
	(0.144)	(0.154)	(0.070)	(0.097)	(0.078)
Deforestation growth _(t-1)_	–	− 0.173***	− 0.131***	− 0.260***	− 0.168***
	–	(0.060)	(0.024)	(0.037)	(0.041)
Urban population (%) growth	–	–	− 7.051**	− 15.874	− 7.290*
	–	–	(3.557)	(22.448)	(3.852)
*Per capita* Energy growth	–	–	− 0.001***	− 0.000***	− 0.010
	–	–	(0.000)	(0.000)	(0.012)
Trade growth	–	–	0.007***	0.008***	0.005
	–	–	(0.001)	(0.001)	(0.004)
Agricultural Employment growth	–	–	0.155	0.052	0.136
	–	–	(0.276)	(0.282)	(0.302)
Constant	0.538***	0.627***	0.456***	0.572***	0.477***
	(0.127)	(0.151)	(0.070)	(0.171)	(0.086)
*N*	2306	2141	1859	1859	1859

Notes: significance levels: *p<0.10, **p<0.05, ***p<0.010. Standard errors are included in parentheses

In Tables 4 and 5, our global data are split into subsamples. Table 4 analyzes the relationship between financial crises and deforestation for four different continents: Africa, America, Asia, and Europe. Results from both OLS and GMM specifications show that financial crises are associated with a decrease in deforestation in Africa, Asia, and Europe, but have no effect on deforestation in America.^2^ Moreover, the magnitude of coefficients varies between continents. The effect is smallest in Europe, with a coefficient equal to − 0.22 to − 0.28, and biggest in Asia, with a coefficient equal to − 0.75 to − 0.83. Note that the OLS and GMM specifications give similar coefficients in terms of sign and magnitude.

**Table 4:** Effect of financial crises on deforestation: continents’ subsamples

Dependent variable: Deforestation growth	(1)	(2)	(3)	(4)	(5)	(6)	(7)	(8)
	OLS specification				GMM specification			
	Africa	America	Asia	Europe	Africa	America	Asia	Europe
Deforestation growth _(t-1)_	− 0.123***	− 0.286***	− 0.104***	− 0.198***	− 0.124**	− 0.269**	− 0.195***	− 0.117***
	(0.029)	(0.044)	(0.027)	(0.021)	(0.054)	(0.124)	(0.055)	(0.042)
Financial Crisis	− 0.472***	− 0.092	− 0.754**	− 0.280***	− 0.427***	− 0.045	− 0.825**	− 0.224***
	(0.161)	(0.108)	(0.351)	(0.088)	(0.158)	(0.132)	(0.414)	(0.072)
Urban population (%) growth	− 18.737*	− 8.322	− 8.260	1.369	− 19.842*	− 4.987	− 7.273	− 0.258
	(11.123)	(7.014)	(8.291)	(15.575)	(11.529)	(6.662)	(8.422)	(14.161)
*Per capita* Energy growth	0.456**	− 0.831	− 0.225	1.035	0.499**	− 0.289	− 0.686*	1.326
	(0.175)	(0.546)	(0.656)	(1.597)	(0.204)	(0.521)	(0.358)	(1.444)
Trade growth	− 0.001	0.113	0.011***	1.044	− 0.088	− 0.068	0.012***	0.920
	(0.322)	(0.358)	(0.003)	(0.939)	(0.373)	(0.467)	(0.003)	(0.761)
Agricultural Employment growth	− 2.474	− 0.818	− 0.044	0.871	− 3.516	− 0.608	− 0.066	0.686
	(1.681)	(1.227)	(0.030)	(0.943)	(2.185)	(1.056)	(0.046)	(1.091)
Constant	0.711***	0.228***	0.416**	0.444***	0.696***	0.139***	0.415**	0.340***
	(0.217)	(0.057)	(0.197)	(0.115)	(0.211)	(0.044)	(0.210)	(0.095)
*N*	530	337	420	477	530	337	420	477

Notes: significance levels: *p < 0.10, **p < 0.05, ***p < 0.010. Standard errors are included in parentheses. Fixed-effects results are available upon request

**Table 5:** Effect of financial crises on deforestation: income-groups’ subsamples

Dependent variable: Deforestation growth	(1)	(2)	(3)	(4)	(5)	(6)	(7)	(8)
	OLS specification				GMM specification			
	High income	Upper middle income	Lower middle income	Low income	High income	Upper middle income	Lower middle income	Low income
Deforestation growth _(t-1)_	− 0.213***	− 0.110***	− 0.103***	− 0.261***	0.049	− 0.137**	− 0.104***	− 0.154*
	(0.024)	(0.022)	(0.028)	(0.027)	(0.103)	(0.069)	(0.025)	(0.083)
Financial Crisis	− 0.274***	− 0.469**	− 0.253	− 0.524**	− 0.182**	− 0.389***	− 0.237	− 0.510**
	(0.082)	(0.188)	(0.158)	(0.189)	(0.086)	(0.148)	(0.176)	(0.213)
Urban population (%) growth	− 27.600*	− 10.957	− 13.247	− 7.069	− 15.774	− 8.455	− 15.306	− 7.021
	(15.118)	(7.875)	(11.978)	(6.122)	(10.269)	(7.011)	(11.389)	(5.163)
*Per capita* Energy growth	− 0.554	0.533***	1.102	− 0.001***	− 1.042	0.424***	0.785	− 0.001
	(0.559)	(0.142)	(0.752)	(0.000)	(0.835)	(0.049)	(0.699)	(0.001)
Trade growth	1.946***	2.252*	0.005***	− 0.325	1.486**	1.130	0.008***	− 0.354
	(0.647)	(1.301)	(0.001)	(0.200)	(0.584)	(0.870)	(0.002)	(0.288)
Agricultural Eployment growth	− 0.062	2.237*	0.178	− 2.919	0.043	2.105	0.157	− 2.618
	(0.069)	(1.290)	(1.427)	(3.196)	(0.107)	(1.332)	(1.910)	(3.470)
Constant	0.439***	0.455***	0.524**	0.564***	0.259***	0.375**	0.535**	0.504***
	(0.084)	(0.150)	(0.214)	(0.150)	(0.081)	(0.151)	(0.225)	(0.150)
N	516	544	475	324	516	544	475	324

Notes: significance levels: *p < 0.10, **p < 0.05, ***p < 0.010. Standard errors are included in parentheses. Fixed-effects results are available upon request.

**Fig. 1:**
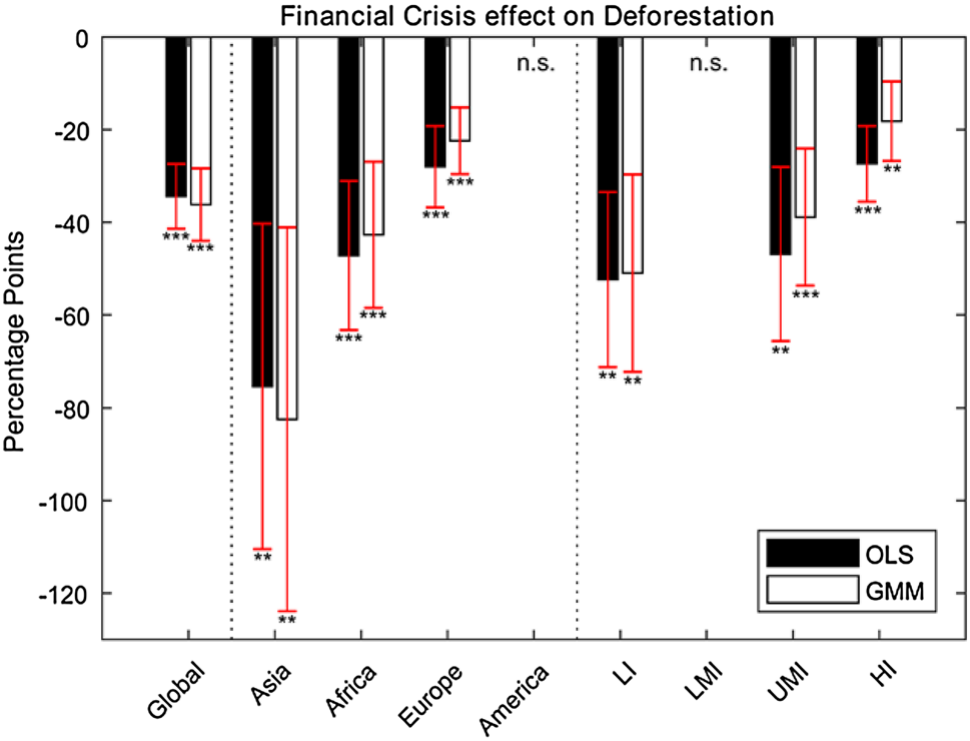
Global and regional effects of financial crises on deforestation using the Global Forest Watch dataset, using the OLS and GMM specifications. Income groups are Low-Income (LI). Lower Middle-Income (LMI), Upper Middle-Income (UMI), and High-Income (HI) countries. Results for America and Low–Middle-Income Countries are not significant (n.s.). Standard errors are included

Table 5 splits the sample into income groups, following the World Bank Atlas Method classification. Results from both OLS and GMM specifications are reported, with very similar coefficients between the two specifications. The negative effect of financial crises on deforestation is confirmed for all income groups, except for lower middle-income countries, whose coefficient is not statistically significant. The magnitude of the reduction in deforestation is inversely related to income: 18 p.p. decrease for high-income countries, 39 p.p. decrease for upper-middle-income countries, and 51 p.p. decrease for low-income countries. Resulting global and regional effects of financial crises on deforestation are graphically presented and summarized in Fig. 1.

### Financial crises effect on roundwood and agricultural commodity production

Changes in agriculture and timber production have been identified as proximate drivers of deforestation, and important channels linked to the relationship between financial crisis and forest loss (see Table 1). Therefore, we test Eqs. (1 and 2) on roundwood and agricultural production, using data from the FAO, and on agricultural land, using data from the ESA-CCI. Results on roundwood production are shown in Table 6, where OLS, Fixed Effects, and GMM specifications are reported. Column 1 includes the crisis variable as the only predictor, column 2 adds the lagged dependent variable, and columns 3–5 include covariates. Results show that financial crises lead to a decrease in roundwood production globally, ranging between − 3.4 and − 6.7 percentage points [no significance for the Fixed Effect model (column 4)]. However, significant results are only at the 10% level, and turn insignificant, in most cases, when splitting the sample into income groups and continents’ sub-samples.^3^

**Table 6:** Effect of financial crises on roundwood production

Dependent variable: Roundwood growth	(1)	(2)	(3)	(4)	(5)
	OLS	OLS	OLS	Fixed effects	GMM
Financial Crisis	− 0.034*	− 0.034*	− 0.064*	− 0.051	− 0.067*
	(0.019)	(0.019)	(0.038)	(0.035)	(0.040)
Roundwood growth_t-1_	–	− 0.002	− 0.002***	− 0.057***	− 0.030***
	–	(0.002)	(0.001)	(0.006)	(0.003)
Urban population (%) growth	–	–	− 3.214	− 0.025	− 3.174
	–	–	(2.238)	(0.958)	(2.154)
*Per capita* Energy growth	–	–	0.106	0.109	0.084
	–	–	(0.154)	(0.150)	(0.137)
Trade growth	–	–	0.000***	0.000***	0.000**
	–	–	(0.000)	(0.000)	(0.000)
Agricultural Employment growth	–	–	− 0.155	− 0.128	− 0.153
	–	–	(0.201)	(0.179)	(0.202)
Constant	0.050***	0.050***	0.101*	0.078***	0.103**
	(0.019)	(0.019)	(0.051)	(0.006)	(0.052)
*N*	7067	7038	3505	3505	3505

Notes: significance levels: *p<0.10, **p<0.05, ***p<0.010. Standard errors are included in parentheses

**Fig. 2:**
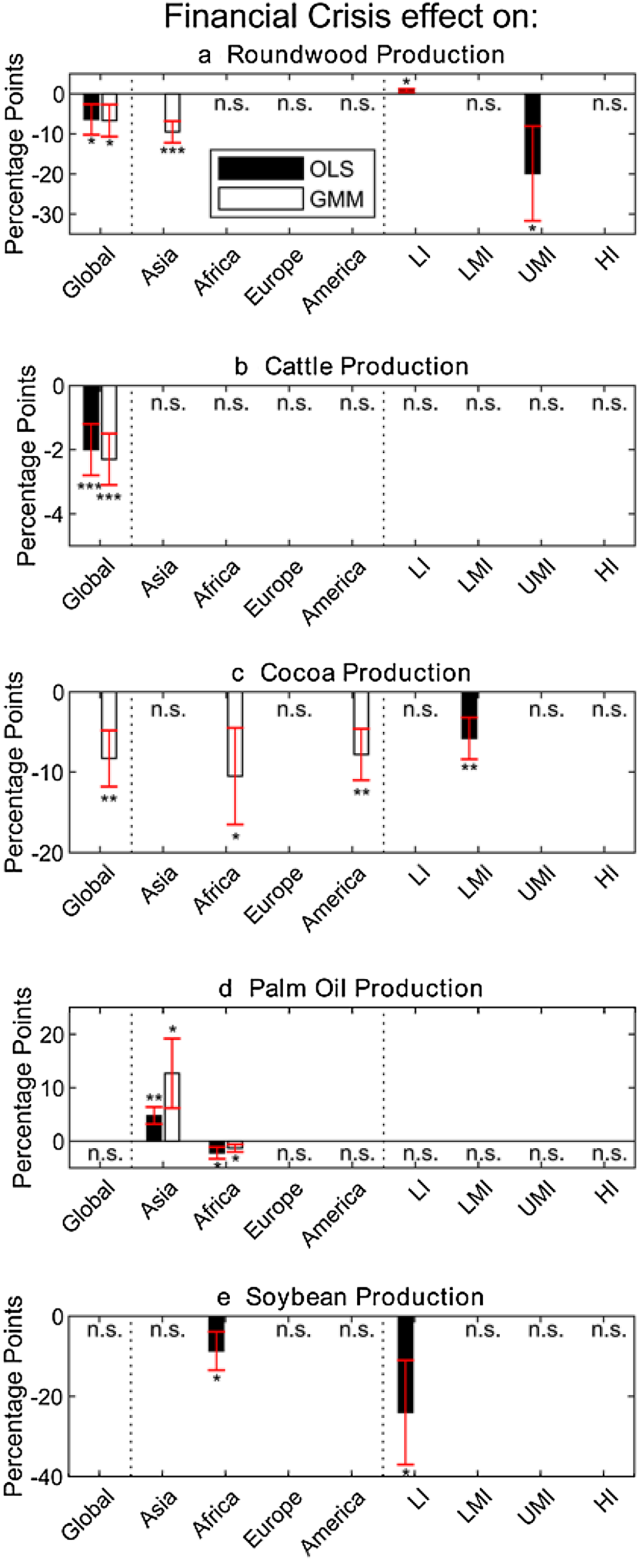
Global and regional effects of financial crises on commodities that contribute largely to deforestation, including roundwood, cattle, cocoa, palm oil, and soybean production (coffee production did not produce significant results). Both OLS and GMM specifications are given. Income groups are Low income (LI), Lower Middle Income (LMI), Upper Middle Income (UMI), and High-Income (HI) countries. Significance levels are shown, while non-significant results are defined as ‘n.s.’. Standard errors are included

Financial crises effects on agricultural production have been calculated based on 5 commodities prevalent in tropical countries—cattle, cocoa, palm oil, coffee, and soybean. Results on two of these commodities, cattle and cocoa production, are given below in Tables 7 and 8, respectively, reporting OLS, Fixed Effects, and GMM specifications. Results for soybean, palm oil, and coffee are not significant at global level so are not presented in tabular format.^4^ Global results show that financial crises are associated with a strongly significant decrease in cattle production globally, ranging between − 1.9 and − 2.3 percentage points (except for the insignificant Fixed Effect model). Furthermore, financial crises affect cocoa production using the GMM specification only (Table 8), with a decrease in cocoa production by 8.3 percentage points with a 5% significance. Income group and continent level results on roundwood production and agricultural commodities are presented in Fig. 2. In most cases, when splitting the sample into income groups and continents’ sub-samples, results become insignificant. Notable exceptions are low and upper mid-income groups for roundwood (Fig. 2a), Africa, America, and lower mid-income groups for cocoa production (Fig. 2c), Asia and Africa for palm oil production (Fig. 2d), and Africa and low-income groups for soybean production (Fig. 2e).

**Table 7:** Effect of financial crises on cattle production

Dependent variable: Cattle Production growth	(1)	(2)	(3)	(4)	(5)
	OLS	OLS	OLS	Fixed effects	GMM
Financial Crisis	− 0.019***	− 0.019***	− 0.020***	− 0.012	− 0.023***
	(0.006)	(0.007)	(0.008)	(0.007)	(0.008)
Cattle Production growth_t-1_	–	− 0.063**	− 0.063	− 0.138***	− 0.164**
	–	(0.027)	(0.038)	(0.037)	(0.076)
Urban population (%) growth	–	–	0.788***	0.538	0.789***
	–	–	(0.240)	(0.651)	(0.271)
*Per capita* Energy growth	–	–	0.002	0.003	0.003
	–	–	(0.004)	(0.004)	(0.010)
Trade growth	–	–	0.000***	0.000***	0.000***
	–	–	(0.000)	(0.000)	(0.000)
Agricultural Employment growth	–	–	− 0.011	− 0.000	− 0.014
	–	–	(0.009)	(0.011)	(0.011)
Constant	0.020***	0.020***	0.016***	0.018***	0.018***
	(0.003)	(0.003)	(0.004)	(0.005)	(0.005)
*N*	3968	3801	3366	3366	3366

Notes: significance levels: *p<0.10, **p<0.05, ***p<0.010. Standard errors are included in parentheses

**Table 8:** Effect of financial crises on cocoa production

Dependent variable: Cocoa Production growth	(1)	(2)	(3)	(4)	(5)
	OLS	OLS	OLS	Fixed effects	GMM
Financial Crisis	− 0.042	− 0.047	− 0.057	− 0.018	− 0.083**
	(0.041)	(0.044)	(0.036)	(0.035)	(0.035)
Cocoa Poduction growth_t-1_	–	− 0.062	− 0.060***	− 0.101***	− 0.490***
	–	(0.048)	(0.019)	(0.021)	(0.067)
Urban population (%) growth	–	–	− 0.279	− 5.911	1.997
	–	–	(1.482)	(4.140)	(2.217)
*Per Capita* Energy growth	–	–	− 0.045***	− 0.039***	− 0.060
	–	–	(0.012)	(0.013)	(0.116)
Trade growth	–	–	− 0.011	-0.029	− 0.034
	–	–	(0.071)	(0.079)	(0.058)
Agricultural Employment growth	–	–	0.371*	0.192	0.320
	–	–	(0.198)	(0.203)	(0.226)
Constant	0.085***	0.094***	0.104***	0.154***	0.073*
	(0.027)	(0.029)	(0.038)	(0.040)	(0.039)
*N*	1212	1161	998	998	998

Notes: significance levels: *p<0.10, **p<0.05, ***p<0.010. Standard errors are included in parentheses

Table 9 shows results from land occupied by agriculture. Coefficients obtained using the OLS specification (columns 1–3) show that crises, on average, are associated with a small positive and significant effect on agricultural coverage. The magnitude of coefficients ranges between 0.001 and 0.002. The coefficient turns insignificant when the Fixed Effects and GMM model are employed. When the sample is split by continents and income groups, the results turn insignificant.^5^

**Table 9:** Effect of financial crises on agricultural coverage

Dependent variable: Aricultural Coverage growth	(1)	(2)	(3)	(4)	(5)
	OLS	OLS	OLS	Fixed effects	GMM
Financial Crisis	0.002**	0.002*	0.001*	0.001	0.001
	(0.001)	(0.001)	(0.001)	(0.001)	(0.001)
Agricultural Coverage growth_t-1_	–	0.250***	0.248***	0.087**	0.346***
	–	(0.066)	(0.042)	(0.038)	(0.051)
Urban population (%) growth	–	–	0.001	0.032	0.011
	–	–	(0.029)	(0.051)	(0.022)
Trade growth	–	–	− 0.000	0.000*	-0.000
	–	–	(0.000)	(0.000)	(0.000)
Constant	0.001***	0.001	0.001	0.001	0.000
	(0.000)	(0.000)	(0.001)	(0.000)	(0.000)
*N*	3867	3698	3522	3522	3522

Notes: significance levels: *p<0.10, **p<0.05, ***p<0.010. Standard errors are included in parentheses

#### Financial crisis effect on ESA-CCI forest cover

In Table 10, we show results coming from the estimation of Eqs. (1 and 2) using forest coverage from ESA-CCI as the dependent variable, as an alternative to Global Forest Watch. The ESA-CCI measures annual change in forest coverage and is available for a longer period of time with respect to GFW deforestation, from 1992 to 2017. Similar to Tables3, 10 presents results from the OLS, Fixed Effects, and GMM specification. Coefficients are, for all specifications except Fixed Effects, negative and statistically significant at the 5% or 1% level, with a magnitude that ranges between 0.001 and 0.002, or 0.1–0.2 percentage points. This indicates that financial crises from 1992 to 2015 are associated with a decrease in forest cover. These results are not in accordance with the ones obtained using Global Forest Watch data, indicating a decrease in deforestation (Table 3; Fig. 1). In the Appendix, we show results obtained by splitting our sample into continents (Appendix Table 14) and income groups (Appendix Table 15). However, coefficients are insignificant for most of the subsamples.

**Table 10:** Effect of financial crises on forest coverage using ESA-CCI data (global sample)

Dependent variable: Forest Coverage growth	(1)	(2)	(3)	(4)	(5)
	OLS	OLS	OLS	Fixed effects	GMM
Financial Crisis	− 0.002***	− 0.002**	− 0.002**	− 0.001	− 0.001**
	(0.001)	(0.001)	(0.001)	(0.001)	(0.001)
Forest Coverage growth_t-1_	–	0.333***	0.330***	0.182***	0.533***
	–	(0.059)	(0.057)	(0.058)	(0.060)
Urban population (%) growth	–	–	− 0.052	− 0.192**	− 0.031
	–	–	(0.034)	(0.083)	(0.020)
*Per capita* Energy growth	–	–	− 0.000	− 0.000	0.000
	–	–	(0.000)	(0.000)	(0.000)
Trade growth	–	–	0.000***	0.000***	0.000
	–	–	(0.000)	(0.000)	(0.000)
Agricultural Employment growth	–	–	0.000	0.000	0.000
	–	–	(0.000)	(0.000)	(0.000)
Constant	0.000	0.000	0.001	0.002**	0.000
	(0.000)	(0.000)	(0.000)	(0.001)	(0.000)
*N*	3937	3765	3396	3396	3396

Notes: significance levels: *p<0.10, **p<0.05, ***p<0.010. Standard errors are included in parentheses

## Discussion

This analysis moves beyond country-specific, case-study, and qualitative assessments of the effect of financial crises on deforestation and forest cover, and is the first to look at the global and regional context of deforestation during financial crisis years, using a panel data approach. This approach also looks at the effects of financial crises on two dominant drivers of deforestation; agricultural commodities and forestry products. From our analysis, financial crises point toward a beneficial effect on reducing deforestation rates for countries in years of crisis. Specifically, financial crises are associated with a global decrease in deforestation rates, with reductions of 36 p.p. (Table 3: GMM specification and Fig. 1). Separating the analysis into continents (Table 4; Fig. 1) showed that financial crises have the largest effect in decreasing deforestation rates in Asia and Africa (− 83 and − 43 p.p.), with a smaller effect in Europe (− 22 p.p.) and no effect in the Americas. Moreover, separating the analysis into income groups (Table 5; Fig. 1), our results show that financial crises have a larger effect on decreasing deforestation rates in low-income countries, than upper middle-income and high-income countries (− 51 vs − 39 and − 18 p.p. respectively). Further investigation into two important channels linked to forest loss points to a negative effect of financial crisis on roundwood production (− 6.7 p.p.), and a negative effect on cattle and cocoa production (− 2.3 and − 8.3 p.p., respectively), albeit at varying significance levels.

These outcomes on deforestation support evidence from case studies on decreases in deforestation, agricultural and timber production during a crisis (Dauvergne 1999; Elliott 2011). Financial crises can lead to decreased timber trade related to decreases in infrastructure (Laurance et al. 2015) and construction (Nilsson 2009; Eurostat 2019; FAO 2020). Financial crises can also lead to a decrease in food prices, and to scarcer capital, trade, and investment for farming and livestock (von Braun 2008; Lin and Martin 2010). This is coupled with the decrease in consumption of meat and sugary products during recessions (Jenkins et al. 2021). Other reasons such as migration away from natural land or mining reductions may also play a part in reducing deforestation rates (Carr 2009; UNECA 2009; Tieguhong et al. 2009). Forest protection during financial crisis may also take place (e.g., Kasa and Naess 2005), with some evidence that demand for Verified Carbon Standards remains strong during financial crises. For instance, there were increases in the volume of voluntary carbon offsets during the Global Financial Crisis by 220% from 2007 to 2008 (Ecosystem Marketplace 2020) dominated by REDD projects (Reduced Emissions from Deforestation and Degradation), with recent pandemic increases of 160% between 2020 and 2021 (Donofrio et al. 2021). REDD projects were prevalent in Latin America and Africa in the years following the Global Financial Crisis (Peters-Stanley et al. 2013).

Using another main yearly dataset on forest cover—the ESA-CCI—we find an overall less significant picture than when using GFW data, where financial crises are associated with a global decrease in forest cover of − 0.1 p.p. (Table 10), an effect driven by Asia (Table 14), and a small positive effect on agricultural land coverage (0.1 p.p.). This outcome on forest and agricultural cover changes from the ESA-CCI provides weak support on financial crises increasing forest loss and agricultural land cover (e.g., Pagiola 2001). It is worth noting that the difference in results using these two datasets (GFW and ESA-CCI) can replicate the broader contradiction regarding reporting to the SDG goals related to forest protection and restoration (Pearce 2018).

### Continental groupings and deforestation drivers

Financial crises are associated with the largest decreases in deforestation rates in Asia (− 83 p.p.; Table 4; Fig. 1). Asia is a significant producer of soybean, cocoa, and cattle, and is the biggest producer of palm oil globally. According to Curtis et al (2018), the largest contributions to deforestation across the continent are commodity-driven agriculture and forestry. Although no significance was found for other agricultural commodities, rates in palm oil production increased during financial crises by 4.8–12.7 p.p. (Fig. 2), and there is a highly significant positive correlation between deforestation and trade in Table 4. This may indicate that during financial crises, palm oil production and exports increased as a way of strengthening foreign reserves, improving balance of payment imbalances, and overall addressing the crises’ adverse affects on livelihoods and the economy. This is in line with evidence from the East Asian crisis were the price of palm oil increased resulting in an expansion of palm production (Pagiola 2001). Yet, deforestation rates decreased during financial crises from 2001 to 2017. A reason for this may be that oil palm may be intensifying in some areas (e.g., in Malaysia: see Varkkey et al. 2018), or oil palm expansion may be happening into non-forested land such as former rubber plantations in Thailand (Saswattecha et al 2016). Another reason may be that Asian roundwood production, where Asian forestry exports are 2nd only to Europe, has strongly decreased during financial crises (− 9.5 p.p.; see Fig. 2a). Elliot (2011) found that demand for timber in Indonesia contracted during the crisis, leading to a reduction in forest exploitation. Chinese exports to the EU also decreased during the Global Financial Crisis (Eurostat 2019).

Yet, it is important to note that GFW may not be able to pick up all spatial changes to the timber industry during a financial crisis. This is because (1) satellites may not detect small-scale degradation or selective logging events; (2) satellites usually include plantations in forest cover products with varying timber felling strategies; (3) logging practices vary in sustainability throughout the world; and (4) forests are dynamic and could involve a mix of management activities. In many lower income countries, wood consumption consists primarily of domestic fuelwood (Mills Busa 2013), meaning that much of wood consumption changes in lower income countries during financial crises will not necessarily be detected by the FAO timber statistics, or the GFW. Illegal logging on the other hand, which in some cases can account for the majority of timber production for both internal and external use, could be detected by the GFW.

Financial crises result in large decreases in deforestation rates in Africa as well (− 43 p.p.; Table 4; Fig. 1). Africa has been dominated by shifting agriculture, of small and medium-scale farmers, as the primary driver of deforestation (Hosonuma et al. 2012; Rudel 2013; Curtis et al. 2018), but there is growing land converted for commodity agriculture, mainly through cocoa in western Africa and oil palm (Ordway et al. 2017). Consequently, our results in Fig. 2 show that financial crises in Africa result in decreased cocoa and palm oil production by 10.5 and 1.3 p.p., respectively. Soybean production in Fig. 2e also shows a decrease of 8.7 p.p. Although African soybean production is only < 1% of the global supply, it is growing rapidly in terms of yield and land area coverage (Cornelius and Goldsmith 2019). The picture with shifting agriculture and financial crises is not clear. Redundancies in other employment sectors and lower capital and technology investment in agriculture can lead to increases in deforestation (Von Braun 2008; Sayer et al. 2012). Others state that small-scale farmers in lower income countries are less affected by crises and could be used as a safety net for food price volatility (De Janvry and Sadoulet 2011). Furthermore, Table 4 shows that urbanization is negatively correlated to deforestation. This may be a result of increased rural–urban migration during financial crises, perhaps due to a decline in timber demand, redundancies in mining (UNECA 2009; Tieguhong et al. 2009), and volatile food prices, all resulting in less pressure on natural land.

Financial crises result in decreasing deforestation rates in Europe (− 22 p.p.; Table 4; Fig. 1), where the principal driver of deforestation in Europe is forestry (Curtis et al. 2018). Forestry in Europe is largely legal with almost 2 million km^2^ of forests under forest certification schemes. The 2008 Global Financial Crisis in Europe led to the levels of both coniferous and deciduous production falling for a number of years, as well as decreased timber imports from tropical countries (Eurostat 2019). This may be the reason for resulting decreases in European deforestation rates, although continental groupings did not show significant effects of financial crisis on roundwood production (Fig. 2a).

Although South America has the largest proportion of agricultural commodity-driven deforestation according to Curtis et al. (2018), there was no significant decrease in deforestation during financial crises (Table 4; Fig. 1), despite a significant decrease in cocoa production and no effect on cattle and soybean production (see Fig. 2c). A reason for this lack in effect on soybean and cattle production may be that South American countries have learned to buffer national and global financial crises through selling to strong foreign markets, e.g., beef and soybean to the Chinese market (Fearnside et al. 2013; Ferchen et al. 2013). Concerning cocoa, much of its production in Central and South America is grown in the forest understory (Somarriba et al. 2013), and/or in Brazilian ‘cabrucas’ or thinned out native-forests agroforestry (Faria et al. 2006), meaning that any change in its production may not directly threaten overstory forest canopies. Over the current pandemic crisis, tropical forests in South America have seen increases in deforestation, where 2020 Brazilian deforestation is the highest since 2008 representing an increase of 47% and 9.5% compared to 2018 and 2019, respectively (Junior et al. 2021). Yet, although there have been reports of increased illegal activity in protected areas and urban-to-rural migration, the World Resources Institute has stated that these increasing do not reveal systematic shifts in forest loss trends that can be clearly link to the pandemic (Weisse and Goldman 2021).

### Income groupings

Our results show that the impact of financial crises on deforestation is contingent on income levels, i.e., during financial crises, deforestation rates drop more in lower-income than upper middle-income and high-income countries [− 51 vs − 39 and − 18 p.p. respectively (Table 5)]. Some of these results may be due to lowering demand for the main drivers of deforestation, where high-income countries are largely dominated by forestry, low-income countries are dominated by shifting agriculture, whereas upper mid-income countries have mixed drivers including forestry, commodity and shifting agriculture, mining, etc. Some of these decreases may be explained by timber and agricultural commodity reductions (Fig. 2). Low-income countries see a significant decline in soybean production (Fig. 2e), although many of these countries are in Africa. Low-income countries also see a small increase in roundwood production (1 p.p. at 10% significance), although this may be an effect of low-income African nations timber trade with Asian economies (e.g., International Institute of Economics and Development 2015). Upper middle-income countries see a large decrease in roundwood production at 20 p.p. (although only at 10% significance), and this includes dominant timber producing countries in East Asia, Southern Africa, and South America.

Furthermore, results from Fig. 1 and Table 5 indicate a larger environmental sensitivity to economic shocks for lower income countries, demonstrated by the larger beneficial effect of financial crisis on deforestation rates in lower income countries. This may link to the evidence that lower income groups have higher deforestation rates than higher income groups (Cropper and Griffiths 1994; Cuaresma et al. 2017), implying that any positive or negative economic change will affect lower income deforestation rates more. Note that our econometric models used in this study seek to establish the contribution of financial crisis on deforeststion, but we note that the over-extraction of environmental resources and loss of forests may contribute to rather than be a consequence of financial crises (e.g., see Harvey 2011; The Guardian 2020) and economic hardship (Srinivasan et al. 2008).

### Comparing GFW and ESA-CCI: data for the Sustainable Development Goals

Evidence on deforestation changes during financial crises is mixed when considering both GFW and ESA-CCI datasets; GFW points toward a decrease in deforestation rates in years of crisis with high significance (Table 3 and Fig. 1), while the ESA-CCI provides weaker support of financial crises increasing forest loss and agricultural land (Tables 9and10). The reasons for this discrepancy could be several. First, the GFW measures yearly deforestation and ESA-CCI measures net forest cover changes, meaning the GFW does not consider forest growth due to reforestation policies, plantation expansion, or natural regeneration of forest. Second, the platforms and spatial resolution of the satellites used are different. GFW uses 30 m Landsat to derive forest loss with canopy cover > 30%. ESA-CCI is provided at 300 m derived from AVHRR, MERIC, SPOT, and PROBA-V, but with different contributions over the 23 year product period, and forest cover from > 15%, to 40% to > 40% tree cover (FAOSTAT 2017; Defourny et al. 2017). This means that at coarser spatial resolutions, many pixels will be a mosaic of cropland/grassland and tree cover, although forest loss and reforestation can originate within these landscapes. Third, a possible explanation for our results is that financial crises may generate two different effects: on one side, a decrease in deforestation due to lower pressure on forests and on economic activities related to them; on the other side, a slowdown in natural regeneration and reforestation projects due to cuts in environmental protection funds (see Table 1).

As stated by the UN in 2018, ‘stopping deforestation and restoring damaged forests could provide up to 30% of the climate solution’ (da Silva et al. 2018). Yet, to achieve the SDGs on forests and carbon (SDG15 and 13), providing more complete global datasets on forests should become an urgent global priority. The current data on yearly deforestation and forest cover come from the GFW and ESA-CCI, with the FAO providing 5 year forest cover. Results from this study and from others (e.g., see Pearce 2018) show that we rely on satellites for our yearly measurements on forest changes, but they are generally incomparable and can provide evidence which can be contradictory. For example, the GFW provides data on deforestation in areas where forests are not permanently lost (e.g., wildfires in Russia and North America), and include plantations and oil palm changes as deforestation, while the ESA-CCI determines many classes of forested and agricultural land, but also classifies mixed land-cover types. Also, recent evidence has shown that considering just the year 2000 baseline forest cover dataset from the GFW was more reliable than the ESA CCI for measuring SDG 15.1.1 over China and India (see Meeuvissen 2020). Considering all of these issues, it is clear that global policy-making initiatives should be focused on producing a consistent, reliable, and freely available dataset informing the SDGs and able to discern (a) yearly deforestation and afforestation/reforestation at high spatial resolution globally; (b) forested disturbance and forest use history; and (c) forest changes in mosaicked landscapes of mixing forests, cropland, grasses, and other land-cover types.

## Conclusion

This study has provided new evidence on the impact of financial crises on deforestation. The analysis used Global Forest Watch data from > 150 countries and > 100 crises in the twenty-first century, and also looked at financial crises on two drivers of deforestation; roundwood and agricultural commodities from the FAO.

Globally, financial crises point toward a beneficial effect on reducing deforestation rates for countries in years of crisis, with reductions of 36 p.p. Financial crises are also associated with a small negative effect on principle drivers of deforestation; roundwood (– 6.7 p.p.), cattle (– 2.3 p.p.), and cocoa production (– 8.3 p.p.), supporting country-level literature on decreases in deforestation and timber production during a crisis (Dauvergne 1999; Elliott 2011).

Financial crises have the largest effect in decreasing deforestation rates in Asia and Africa (– 83 and – 43 p.p.), with a smaller effect in Europe (– 22 p.p.) and no effect in the Americas. Drivers behind these effects may be different, from forestry reductions in Europe, to palm oil (– 1.3 p.p.), cocoa (– 10.5 p.p.), and soybean (– 8.7 p.p.) reductions in Africa, to a combination of timber (– 9.5 p.p) and commodity agriculture changes (e.g., palm oil) in Asia. Moreover, financial crises have a larger effect on decreasing deforestation rates in low-income countries, than upper middle-income and high-income countries (– 51 vs – 39 and – 18 p.p. respectively), indicating a larger environmental sensitivity to economic shocks for lower income countries.

Using the yearly and global ESA-CCI forest cover dataset, we find that financial crises lead to a global decrease in forest cover of – 0.1 p.p., which points to financial crises increasing forest loss and agricultural land cover (e.g., Pagiola 2001). These opposite results between the GFW and ESA-CCI present a big challenge and constraint in studying forests and understanding their relationship with economic slowdowns. To achieve the SDG goals related to forests, we urgently need better global forest cover data with better forest loss/gain data, disturbance history, and understanding of mosaicked landscape dynamics within a satellite pixel. Furthermore, future research into determining the causality between deforestation during financial crises and social, economic, and environmental variables will provide insight into global and regional-level drivers of environmental change. Determining causality using methods like Granger causality (e.g., Zambrano-Monserrate et al. 2018; Nathaniel and Bekun 2020), may also begin to provide a causal link between environmental degradation and financial crises (see Harvey 2011; The Guardian 2020). National and sub-national impacts of financial crises on deforestation may also prove important as the impacts of economic shocks are not felt equally by all regions within a country (OECD 2020).

Forests constitute critical transition zones for generating synergies that can help us meet the SDGs and transition to sustainability (see Alcamo et al. 2020), especially in a period of heightened global economic vulnerabilities (Antoniades and Griffith‐Jones 2018). Our results suggest that reductions in deforestation rates during periods of financial crises could be taken as an opportunity by governments to enhance their sustainable management of forested landscapes during a period of commodity production downturn (Burns et al. 2019). Otherwise, the beneficial effects of financial crises on forests may be lost quickly once a crisis finishes, where environmental policy ambitions and activism may wane and slip down national agendas. Maintaining the climate and sustainable development agenda is critical in the beginning of the 2020s with less than 10 years left to achieve the Sustainable Development Goals. With the Coronavirus pandemic, we have seen again that a reduction in economic activity can be temporarily beneficial for certain environment criteria such as air pollution and greenhouse gas emissions (Antonarakis 2020). Yet, the UN has stated that the pandemic has potentially reversed progress with land degradation continuing, massive numbers of species risking extinction, and unsustainable production and consumption (UN 2020). Furthermore, COVID-19 recovery packages are pledging around 20% to green recovery, but only 0.4% ($56.3 billion) on natural capital and ecosystem protection (O’Callaghan et al. 2020; Antoniades et al. 2022).

Sustainable Development initiatives such as Zero-Deforestation Commitments from producers and traders (Humphreys et al. 2019) and the New York Declaration on Forests have advocated for the decoupling of forest loss and commodity production, with varying degrees of success (Haupt et al. 2017; Lambin et al. 2018). Decoupling food production (SDG2) and forest ecosystems and management (SDG15 and 12) with the help of zero deforestation commitments across NGOs, private sector, international organizations, and grass root organizations (SDG17) are necessary in achieving synergies across the Sustainable Development Goals so as to reach a sustainable global socio-environmental path.

## Appendix

See Tables 11, 12, 13, 14, 15.

**Table 11:** List of countries included in our analysis

Albania	Comoros	Haiti	Mauritius	Slovak republic
Algeria	Congo, D.R	Honduras	Mexico	Slovenia
Angola	Congo, R	Hungary	Moldova	South Africa
Argentina	Costa Rica	Iceland	Mongolia	South Sudan
Armenia	Côte d’Ivoire	India	Morocco	Spain
Australia	Croatia	Indonesia	Mozambique	Sri Lanka
Austria	Cyprus	Iran, I.R. of	Myanmar	St. Kitts and Nevis
Azerbaijan	Czech Republic	Ireland	Namibia	Sudan
Bangladesh	Denmark	Israel	Nepal	Suriname
Barbados	Djibouti	Italy	Netherlands	Swaziland
Belarus	Dominica	Jamaica	New Caledonia	Sweden
Belgium	Dominican Rep	Japan	New Zealand	Switzerland
Belize	Ecuador	Jordan	Nicaragua	Syria
Benin	Egypt	Kazakhstan	Niger	Tajikistan
Bhutan	El Salvador	Kenya	Nigeria	Tanzania
Bolivia	Equatorial Guinea	Korea	Norway	Thailand
Bosnia Herz	Eritrea	Kuwait	Pakistan	Togo
Botswana	Estonia	Kyrgyz Republic	Panama	Trinidad and Tobago
Brazil	Ethiopia	Laos	Papua New Guinea	Tunisia
Brunei	Fiji	Latvia	Paraguay	Turkey
Bulgaria	Finland	Lebanon	Peru	Turkmenistan
Burkina Faso	France	Lesotho	Philippines	Uganda
Burundi	Gabon	Liberia	Poland	Ukraine
Cambodia	Gambia, The	Libya	Portugal	United Kingdom
Cameroon	Georgia	Lithuania	Romania	United States
Canada	Germany	Luxembourg	Russia	Uruguay
Cape Verde	Ghana	North Macedonia	Rwanda	Uzbekistan
Central African R	Greece	Madagascar	São Tomé & Principe	Venezuela
Chad	Grenada	Malawi	Senegal	Vietnam
Chile	Guatemala	Malaysia	Serbia	Yemen
China	Guinea	Maldives	Seychelles	Yugoslavia, SFR
China: Hong Kong	Guinea-Bissau	Mali	Sierra Leone	Zambia
Colombia	Guyana	Mauritania	Singapore	Zimbabwe

**Table 12:** Unit root test based on augmented Dickey–Fuller tests, 1 lag

Dependent variables	*p* statistic	*p* value
Forest loss	571.36	0.0000
Tree Coverage area from CCI	442.79	0.0002
Roundwood	278.70	0.9876
Cattle Production	664.50	0.0000
Agricultural Land from CCI	503.82	0.0000
Cocoa Production	754.35	0.0000
**Independent variables**		
Energy *per capita*	323.5512	0.6197
Trade	612.0025	0.0000
Urban Population %	611.8155	0.0000

**Table 13:** Effect of global financial crisis of 2008 on deforestation

Dependent variable: Deforestation growth	(1)	(2)	(3)	(4)
	OLS	OLS	Fixed Effects	GMM
Global Financial Crisis 2008	− 0.434***	− 0.202***	− 0.164*	− 0.193***
	(0.138)	(0.070)	(0.094)	(0.068)
Deforestation growth _(t-1)_	–	− 0.128***	− 0.258***	− 0.154***
	–	(0.024)	(0.037)	(0.042)
Urban population (%) growth	–	− 6.256*	− 16.242	− 6.263*
	–	(3.457)	(22.558)	(3.654)
*Per capita *Energy growth	–	− 0.001***	− 0.000***	− 0.009
	–	(0.000)	(0.000)	(0.012)
Trade growth	–	0.004***	0.005***	0.002
	–	(0.001)	(0.001)	(0.004)
Agricultural Employment growth	–	0.148	0.042	0.137
	–	(0.272)	(0.280)	(0.299)
Constant	0.539***	0.440***	0.558***	0.453***
	(0.127)	(0.068)	(0.168)	(0.082)
*N*	2306	1859	1859	1859

Notes: Significance levels: **p* < 0.10, ***p* < 0.05, ****p* < 0.010. Standard Errors are included in parentheses. Although these results cover all countries globally, the “Global Financial Crisis 2008” dummy variable is equal to one only for those that experienced the Global Financial Crisis in 2008 as reported in Laeven and Valencia (2018). The variable stays equal to one for the whole duration of the crisis, corresponding to the period 2008–2012 for most countries

**Table 14:** Effect of financial crises on forest coverage using ESA-CCI: continents subsamples

Dependent variable: Forest Coverage Growth	(1)	(2)	(3)	(4)	(5)	(6)	(7)	(8)
	OLS specification				GMM specification			
	Africa	America	Asia	Europe	Africa	America	Asia	Europe
Forest Coverage growth_t-1_	0.278***	0.335**	0.364***	0.427***	0.417***	0.448	0.529***	0.478***
	(0.085)	(0.146)	(0.098)	(0.060)	(0.075)	(0.371)	(0.042)	(0.089)
Financial Crisis	− 0.003	− 0.002	− 0.002*	− 0.000	− 0.003	− 0.002	− 0.002*	− 0.000
	(0.003)	(0.002)	(0.001)	(0.000)	(0.002)	(0.001)	(0.001)	(0.000)
Urban population(%) growth	− 0.062	0.037	− 0.085*	0.062	− 0.044	0.033	− 0.063*	0.055
	(0.080)	(0.047)	(0.048)	(0.051)	(0.050)	(0.038)	(0.037)	(0.046)
*Per capita* Energy growth	− 0.000	− 0.002	− 0.003	− 0.001	− 0.000	-0.003	0.000	0.000
	(0.000)	(0.002)	(0.004)	(0.003)	(0.000)	(0.003)	(0.005)	(0.003)
Trade growth	− 0.000	0.003	0.000	− 0.002	0.001	0.002	0.000***	− 0.005**
	(0.005)	(0.002)	(0.000)	(0.002)	(0.008)	(0.003)	(0.000)	(0.002)
Agricultural Employment growth	0.000	− 0.003	0.001***	0.000	0.001	− 0.003	0.001*	− 0.001
	(0.005)	(0.006)	(0.000)	(0.002)	(0.005)	(0.005)	(0.000)	(0.003)
Constant	0.001	− 0.001	0.001	− 0.000	0.000	− 0.001	0.001	− 0.000
	(0.001)	(0.001)	(0.001)	(0.000)	(0.001)	(0.001)	(0.001)	(0.000)
*N*	919	571	850	805	919	571	850	805

Notes: Significance levels: **p* < 0.10, ***p* < 0.05, ****p* < 0.010. Standard errors are included in parentheses

**Table 15:** Effect of financial crises on forest coverage using ESA-CCI: income-group subsamples

Dependent variable: Forest Coverage growth	(1)	(2)	(3)	(4)
	GMM specification			
	High income	Upper middle income	Lower middle income	Low income
Forest Coverage growth_t-1_	0.547***	0.256	0.631***	0.427***
	(0.159)	(0.166)	(0.110)	(0.072)
Financial Crisis	− 0.000	− 0.000	− 0.001	− 0.002
	(0.000)	(0.001)	(0.001)	(0.002)
Urban population (%) growth	0.042	0.007	0.002	− 0.060*
	(0.069)	(0.050)	(0.022)	(0.033)
*Per capita* Energy growth	− 0.001	0.000	− 0.001	− 0.001
	(0.003)	(0.000)	(0.003)	(0.004)
Trade growth	− 0.003	0.000***	− 0.000	0.003
	(0.005)	(0.000)	(0.000)	(0.009)
Agricultural Employment growth	0.000	− 0.002	− 0.009	− 0.008
	(0.001)	(0.002)	(0.007)	(0.013)
Constant	0.000	0.000	− 0.000	0.001*
	(0.000)	(0.001)	(0.000)	(0.001)
*N*	1037	972	793	594

Notes: Significance levels: **p* < 0.10, ***p* < 0.05, ****p* < 0.010. Standard errors are included in parentheses

## Footnotes

1 Unit root test results are presented in Appendix Table 12 in the Appendix.

2 We also ran separate regressions for North and South America, finding that the effect is null for both areas. Results are available from the authors upon request.

3 Results are available from the authors upon request.

4 Results are available from the authors upon request.

5 Results are available from the authors upon request.
